# Central pain modulatory mechanisms of attentional analgesia are preserved in fibromyalgia

**DOI:** 10.1097/j.pain.0000000000002319

**Published:** 2021-05-01

**Authors:** Valeria Oliva, Robert Gregory, Jonathan C.W. Brooks, Anthony E. Pickering

**Affiliations:** a School of Physiology, Pharmacology & Neuroscience, University of Bristol, Bristol, United Kingdom; b Anaesthesia, Pain & Critical Care Sciences, Bristol Medical School, University Hospitals Bristol, Bristol, United Kingdom; c School of Psychological Science, University of Bristol, Bristol, United Kingdom; d University of East Anglia Brain Imaging Centre, School of Psychology, Norwich, United Kingdom

**Keywords:** Fibromyalgia, Pain, fMRI, Attention, Brainstem, Analgesia

## Abstract

Fibromyalgia is a prevalent pain condition that is associated with cognitive impairments including in attention, memory, and executive processing. It has been proposed that fibromyalgia may be caused by altered central pain processing characterised by a loss of endogenous pain modulation. We tested whether attentional analgesia, where cognitive engagement diminishes pain percept, was attenuated in patients with fibromyalgia (n = 20) compared with matched healthy controls (n = 20). An individually calibrated, attentional analgesia paradigm with a 2 × 2 factorial design was used with brain and brainstem-focussed functional magnetic resonance imaging. Patients with fibromyalgia had both lower heat pain thresholds and speeds in a visual attention task. When this was taken into account for both attentional task and thermal stimulation, both groups exhibited an equivalent degree of attentional analgesia. Functional magnetic resonance imaging analysis showed similar patterns of activation in the main effects of pain and attention in the brain and brainstem (with the sole exceptions of increased activation in the control group in the frontopolar cortex and the ipsilateral locus coeruleus). The attentional analgesic effect correlated with activity in the periaqueductal gray and rostral ventromedial medulla. These findings indicate that patients with fibromyalgia can engage the descending pain modulatory system if the attentional task and noxious stimulus intensity are appropriately titrated.

## Abstract 2

Supplemental Digital Content is Available in the Text.

The use of brainstem-optimised functional magnetic resonance imaging and a calibrated attentional analgesia task demonstrates that patients with fibromyalgia can recruit the descending pain modulatory system like healthy controls.

## 1. Introduction

Fibromyalgia is a common, chronic condition characterised by widespread pain with hyperalgesia in muscles and joints without any identifiable alternative causative pathology.^6,70,84^ In addition to widespread pain, fibromyalgia is syndromically linked to fatigue, sleep deficits, and difficulties in concentration, an array of symptoms, which has been referred to as “fibrofog”.^43,81^ A single underlying pathophysiological cause for fibromyalgia is yet to be fully elucidated,^69^ and the current diagnostic criteria are based on self-reported measures.^75,82,83^

There are a plethora of studies reporting alterations in nociception and pain processing in patients with fibromyalgia. One intriguing line of investigations has reported a small fibre deficit and altered function of nociceptive primary afferents^31,50,56,72,77^ which may give rise to hyperalgesia. As a counterpoint theory, fibromyalgia has also been proposed to be a “centralised” pain condition^12^ characterised by augmented brain responses to noxious stimuli that underlies hyperalgesia.^15,30,66^ In support of a central aetiology of fibromyalgia, there have been reports of impairments in endogenous pain modulatory mechanisms, such as conditioned pain modulation^8,42,49^ and exercise-induced analgesia.^80^ This has, in part, been the justification for the use of treatments to boost central pain modulatory circuits through the use of monoaminergic reuptake inhibitors (increasing noradrenaline and serotonin), which are among the few medications with any evidence of efficacy in fibromyalgia.^7,12^

Endogenous pain modulation^60^ can also be engaged by cognitive manipulations, such as placebo analgesia^4,19^ or a shift in attentional focus.^3,78^ In healthy subjects, attentional analgesia has been shown to involve brainstem structures such as the rostral ventromedial medulla (RVM), locus coeruleus (LC), and periaqueductal gray (PAG)^9,59,76,78^ that mediate a component of their pain modulatory effects through endogenous monoamines.^53,60^ These brainstem regions are intrinsically challenging to image^11^ and have been only sparsely investigated in fibromyalgia despite being implicated as part of the causative central pathology.

The known link between fibromyalgia and impaired cognitive performance in domains such as attention, memory, and executive processing^16,26,29,68^ provides a rationale to investigate a form of endogenous analgesia that is driven by cognitive focus, ie, attentional analgesia. We hypothesised that there would be a demonstrable deficiency in attentional analgesia in patients with fibromyalgia and further that whole-brain/brainstem-optimised functional magnetic resonance imaging (fMRI) could determine where any deficit originated within the descending pain modulatory system or the attentional network.

## 2. Methods

The study had ethical approval from the NHS South Central Oxford B Research Ethics Committee (reference 13/SC/0617). All subjects gave written informed consent for study participation. The study was undertaken in the Clinical Research and Imaging Centre at the University of Bristol (CRiCBristol).

### 2.1. Recruitment

Patients with fibromyalgia were recruited from local pain management clinics by clinician referral and poster advertisements. Sex-matched, healthy control subjects were recruited using poster and email advertisements at the University of Bristol. All subjects were screened for participation by telephone before attending for their single session. To meet the inclusion criteria, they required a confirmed clinical diagnosis of fibromyalgia for at least 6 months before entry into the study. Subjects were excluded if they had other chronic painful conditions, were pregnant, or had a history of neurological or major psychiatric illness. In addition, for control subjects, the presence of significant medical disorder precluded participation. Standard safety inclusion/exclusion criteria for participation in MRI studies were also applied. All subjects completed the Widespread Pain and Symptom Severity Index^82^ to validate the fibromyalgia diagnosis for patients and to confirm the absence of fibromyalgia symptoms for control subjects.

A total of 54 subjects (32 patients and 22 controls) were screened for the study, of which 14 failed the screening (3 were left-handed, 9 were unable to attend, 1 was unable to lie flat in the scanner, and 1 did not pass the MRI screening). Twenty right-handed patients with fibromyalgia (mean age 43, range 25-60; 18 women) and 20 right-handed, healthy subjects (mean age, 35 years, range 20-59 years; 18 women) participated in the study. The healthy control subjects were 8 years younger on average than the patients with fibromyalgia (*t* test, *P* = 0.03). Patients were not required to alter their regular medications which included non-opioid analgesics (n = 13), opioids (n = 9), tricyclic antidepressants/serotonin and noradrenaline reuptake inhibitors (n = 11), and gabapentinoids (n = 7).

### 2.2. Experiment

Written informed consent was taken, and MRI safety questionnaires were completed on the day of study. The subjects were told that the experiment was to examine the interaction between pain and attention in the brain with no mention of the phenomenon of attentional analgesia to avoid generating an expectation about the study purpose. The American College of Rheumatology (ACR) Widespread Pain and Symptom Severity Index^82^ was completed with the assistance of clinician experimenters. Assessments were also made using the Edinburgh Handedness Inventory^58^, PainDETECT,^24^ the “pain now” and “pain on average” scales from the Brief Pain Inventory,^13^ Hospital Anxiety and Depression scale,^87^ and Pain Anxiety Symptom Scales^52^. Any medications taken in the 72 hours before the session were recorded for all participants.

Both groups had a thermal quantitative sensory testing (QST) with a circular contact thermode (CHEPS Pathway, MEDOC, Israel) applied on the left volar forearm using a modified version of the standardised protocol and script^67^ (that included warm detection threshold, heat pain threshold, cold detection threshold, and cold pain threshold). Study participants also had pressure pain threshold assessment over the thenar eminence using an algometer (Somedic, Sweden). After a short comfort/snack break, participants moved on to the calibration for the fMRI experiment.

The experimental protocol was identical in structure to the one described in our previous studies.^9,59^ In brief, participants received thermal stimuli to their left forearm for 30 seconds at either 36°C (low temperature) or 42 to 45°C (high temperature), and a pseudorandom series of 1-second long “spikes” of 2, 3, or 4°C above these temperatures were superimposed to minimise habituation to stimulation. The high-temperature stimulus was calibrated for each individual to identify the thermal stimulus that produced a 6 of 10 pain score.

Participants were also calibrated for a rapid serial visual presentation (RSVP) attentional task,^64^ where they were presented with rapidly changing letters and numbers on a display screen and they were instructed to press a button when spotting the number 5. The task had 2 possible levels of difficulty (easy or hard). The task was individually titrated such that its speed of presentation (ie, interstimulus interval [ISI]) was performance matched to ability. Each participant's task performance was assayed over a range of ISIs (from 32 to 256 ms) by calculating d-prime (d'). The d’ values were fitted with a sigmoidal function and used to estimate the presentation speed corresponding to a 70% task performance which was used for their hard task during the experiment.

The ISI for the easy task was set to

(1) 192 ms if the subject's hard task ISI was < 96 ms,
(2) 256 ms if the hard task ISI was ≥ 96 and < 256 ms, and
(3) 384 ms if the hard task ISI was = 256 ms.

The fMRI experiment had a 2 × 2 factorial design with 4 combinations for task and temperature (easy|high, hard|high, easy|low, and hard|low) and has been described in detail previously.^9,59^ Each experimental block lasted 70 seconds (comprising a fixation period with only a cross on the screen [17 s], brief instruction to spot the target among distractors [5 s], RSVP task performance and concurrent thermal stimulus [30 s], a further fixation period [10 s], and finally a rating period to obtain the pain score [8 s]). The blocks were presented in a pseudorandom sequence within sessions and across participants. Each combination was repeated 4 times giving a total of 16 blocks. Task performance (hits, misses, and false alarms) was also recorded during the experiment.

### 2.3. Magnetic resonance imaging data acquisition

Brain images were acquired with a 3T Siemens Skyra whole-body MR system using the same acquisition sequences as our previous studies.^9,59^ In brief, subjects' heads were positioned within the 32-channel receive only head coil and memory foam pads placed around the skull to help minimise movement. After acquisition of localiser images, a sagittal T1-weighted MPRAGE volumetric scan was acquired with TE/TI/TR = 2.28/1000/2200 ms, flip angle = 9° and a resolution of 0.86 x 0.86 x 0.86 mm, phase encoding direction = A-P, and GRAPPA acceleration factor = 2. Functional imaging data were acquired with an echo planar imaging (EPI) sequence and GRAPPA acceleration factor = 2, TE/TR = 30/3000 ms, flip angle = 90°, and a resolution of 1.77 × 1.77 × 3.5 mm. Finally, to correct image distortion in EPI data, a gradient echo field map was acquired with TE1/TE2/TR = 4.92/7.38/520 ms, flip angle 60°, and a resolution of 3 × 3 × 3.5 mm. During the fMRI experiment, cardiac and respiratory waveforms were recorded using a pulse oximeter and respiratory bellows for subsequent physiological noise modelling.^11^

### 2.4. Functional magnetic resonance imaging analysis

Functional images were preprocessed and analysed in FEAT (FSL version 6^39^). The preprocessing pipeline was consistent with our previous articles^9,59^ and included motion correction with MCFLIRT,^38^ fieldmap unwarping with FUGUE,^37^ registration to standard MNI template with FNIRT^1^ and FLIRT,^40^ and 4-mm spatial smoothing and high-pass temporal filtering using a 90-second cutoff. The general linear model (GLM) in FEAT, part of FSL, was used to assess brain activation to the 4 experimental conditions (easy|high, hard|high, easy|low, and hard|low) and nuisance regressors (task instruction and rating periods), which were convolved with a hemodynamic response function. The design also included temporal derivatives, local autocorrelation correction (FILM^85^), and a set of regressors modelling physiological noise.^10,33^

Simple main effects were estimated by first creating difference contrast images between conditions at the first (ie, subject) level, for example, (easy|high + hard|high) - (easy|low + hard|low) for the main effect of temperature, looking for regions more active during high temperature stimulation irrespective of task difficulty. Note that the reverse contrast was also calculated. This process was repeated for the simple main effects of task, along with the interaction contrasts. Next, these difference contrast images were passed up to the second (ie, group) level where 1-sample *t* tests were used for statistical inference (for pooled data) and 2-sample *t* tests (for estimation of group differences). For consistency, the same approach was used for both whole-brain analysis with FEAT and for masked analysis using RANDOMISE. The analysis approach taken is recommended by the developers of the FSL software package because the GLM is not designed to model repeated measures in 2 × 2 factorial designs. Whole-brain group differences were assessed with a 1-sample *t* test in FEAT using a mixed-effects model (FLAME) and cluster-based correction for multiple comparison (with cluster-forming threshold Z > 3.1 and cluster-corrected *P* < 0.05 to adjust for family-wise error, in accordance with the latest recommendations for spatial analysis of fMRI data^21^).

The brainstem-focussed analysis was performed at the group level using a set of anatomical masks and statistical inference using permutation testing^55^ in RANDOMISE (part of FSL). This analysis used predefined regions of interest based on previously defined probabilistic masks of the a priori specified brainstem nuclei (PAG, RVM, and left/right LC, defined previously^9^). A 2-sample unpaired *t* test design was built with GLM (in FSL) in accordance with FEAT guidance. The number of permutations was set to 10,000 in line with guidelines,^21^ and results were reported using threshold-free cluster enhancement correction *P* < 0.05.

Where simple main effects or interactions were found in the imaging data, the nature of these differences was explored using FEATQUERY. Parameter estimates were extracted from each experimental condition (ie, easy|low vs rest, hard|low vs rest, easy|high vs rest, and hard|high vs rest), and their relationship to the individual behavioural responses was examined.

The magnitude of attention-mediated analgesia was compared with BOLD signal change in the brainstem nuclei (PAG, RVM, and LC) specified a priori (as per our earlier study^9^). Average pain ratings obtained during high-temperature stimulation at the 2 different task difficulties were subtracted (ie, easy|high - hard|high) and demeaned to obtain a group-level covariate. The difference in the BOLD signal recorded for hard|high minus easy|high was correlated with the difference in pain ratings in an inter-subject parametric regression model. RANDOMISE was used to assess correlations in PAG, RVM, and left and right LC masks. The latter analysis was performed on the whole cohort (patients with fibromyalgia and healthy controls).

All whole-brain results (group means and group comparisons) are reported for Z > 3.1, cluster-corrected *P* < 0.05. All brainstem results are reported for *P* < 0.05, threshold-free cluster enhancement corrected.

### 2.5. Questionnaire, quantitative sensory testing, and behavioural data analysis

All statistical analyses (questionnaires, QST, pain ratings, and task performance) were performed in SPSS (version 26). Unpaired t tests were used on questionnaire results to detect differences between patient and control groups.

Hit rate (the proportion of correct responses to targets) and false alarm rate (the proportion of responses to nontargets) were calculated and z transformed. Subsequently, *d’* was calculated as the difference between z transformed hit rate and z transformed false alarm rate. The interstimulus intervals were compared with a Mann–Whitney *U* test.

Pain ratings and task performance recorded during the fMRI experiment were analysed with a mixed analysis of variance (ANOVA) (with 2 within-subject factors: task and temperature and 1 between-subjects factor: group). A prespecified post-hoc comparison of the difference in pain scores between the easy|high vs hard|high condition was undertaken to identify any attentional analgesic effect.

Before statistical analysis, data were examined for the presence of outliers, normality of distribution, and equality of variance. The results are reported as mean ± SD or median and range where appropriate. The indicative significance level was set to *P* < 0.05 throughout.

## 3. Results

### 3.1. Demographics

All patients met the ACR 2010 diagnostic criteria for fibromyalgia,^82^ scoring 13.5 ± 2.6 on the Widespread Pain Index (WPI) and 10.0 ± 1.5^7–12^ on the Symptom Severity (SS) Scale score (WPI ≥7 and SS ≥5, Table 1). None of the healthy controls met the ACR 2010 diagnostic criteria, scoring 1.0 ± 1.0 [0-3] on the WPI and 2.0 ± 1.1^1–4^ on the SS (Table 1).

**Table 1:** Results of questionnaires from patients with fibromyalgia and healthy controls.

Questionnaire	Patients with fibromyalgia	Healthy controls	Significance
Widespread Pain Index (ACR)	13.5 ± 2.6	1.0 ± 1.0	N/A
Symptom Severity (ACR)	10 ± 1.5	2 ± 1.1	N/A
Pain now (BPI)	5.3 ± 1.6	0.1 ± 0.2	*P* < 0.0001
Pain on average (BPI)	6.4 ± 1.7	0.7 ± 1.0	*P* < 0.0001
PainDETECT	15.7 ± 8.2	2.4 ± 3.3	*P* < 0.0001
Hospital Anxiety (HADS)	12.2 ± 3.6	4.6 ± 4.0	*P* < 0.0001
Hospital Depression (HADS)	10.5 ± 4.7	1.3 ± 1.3	*P* < 0.0001
Pain anxiety symptom (cognitive)	18.4 ± 4.3	5.3 ± 6.6	*P* < 0.0001
Pain anxiety symptom (avoidance)	14.6 ± 5.6	5.8 ± 5	*P* < 0.0001
Pain anxiety symptom (fear)	11.2 ± 6.8	1.6 ± 1.9	*P* < 0.0001
Pain anxiety symptom (anxiety)	11.6 ± 5.5	1.5 ± 2.4	*P* < 0.0001

All comparisons with unpaired *t* test with the exception of PAS which is a 1-way ANOVA with Sidak post-hoc tests.

ACR, American College of Rheumatology; BPI, Brief Pain Inventory; HADS, Hospital Anxiety and Depression Scale.

As expected, the patients with fibromyalgia had higher ratings than the control group for the “pain now” (5.3 ± 1.6 vs 0.1 ± 0.2, respectively, *P* < 0.0001) and “pain average” (6.4 ± 1.7 vs 0.7 ± 1.0, respectively, *P* < 0.0001, Table 1) domains of the Brief Pain Inventory. They also scored higher on the PainDETECT questionnaire compared with controls (15.7 ± 8.2 vs 2.4 ± 3.3, respectively, *P* < 0.0001, Table 1). Patients with fibromyalgia had elevated anxiety and depression scores (12.2 ± 3.6 and 10.5 ± 4.7, with 17 and 15 patients scoring >8, respectively) in comparison with healthy controls (4.6 ± 4.0 and 1.3 ± 1.3, with 3 scoring >8 for anxiety) on the Hospital Anxiety and Depression Scale (*P* < 0.0001 in both cases, Table 1). Patients with fibromyalgia also had higher scores in the cognitive, avoidance, fear, and anxiety sections of Pain Anxiety Symptom Scales (all *P* < 0.0001, Table 1).

### 3.2. Quantitative sensory testing

**Figure 1.**
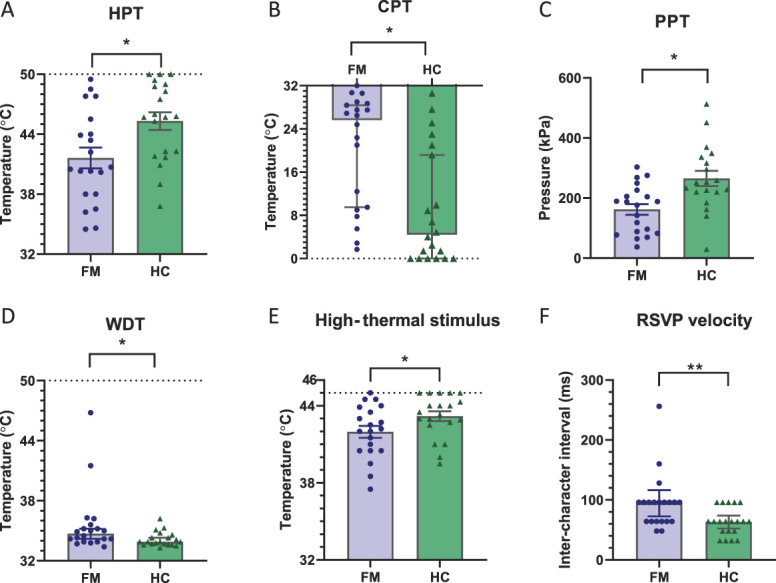
Quantitative sensory testing and calibration. Quantitative sensory testing showed that patients with fibromyalgia had smaller (A) heat pain thresholds, (B) cold pain threshold, and (C) pressure pain threshold. (D) Patients with fibromyalgia also had an elevated warm detection threshold. (E) The thermode temperature used for the high-thermal stimulus was lower in patients with fibromyalgia. (F) The inter-character interval for the RSVP task was longer in the patients with fibromyalgia. Data presented as mean ± SEM and comparison between groups with unpaired *t* test except for (C–E) which are median [IQR] and analysed with the Mann–Whitney test (*P < 0.05, **P < 0.01). IQR, interquartile range; RSVP, rapid serial visual presentation.

Patients with fibromyalgia exhibited hyperalgesia to thermal and deep pressure stimuli when compared with controls. The heat pain threshold was lower in patients with fibromyalgia (41.6 ± 4.6°C fibromyalgia vs 45.3 ± 3.9°C controls, *P* = 0.01, unpaired *t* test, Fig. 1A), and the cold pain threshold was at a higher temperature (fibromyalgia 25.7°C [1.7-32°C] vs healthy controls 4.5°C [0-30.6°C], *P* = 0.001, Mann–Whitney test, Fig. 1B). Similarly, the pressure pain threshold was lower in patients with fibromyalgia (fibromyalgia 162 ± 18 vs control 265 ± 25 kPa, *P* = 0.0019, unpaired *t* test, Fig. 1C). The warm detection threshold was higher in patients with fibromyalgia (34.7°C [33.4-46.8°C] vs 33.9°C [33.3-36.2°C], *P* = 0.016, Mann–Whitney U test Fig. 1D). There were 2 outliers in the fibromyalgia group, and their exclusion reduced the difference in medians to 0.5°C, but the result remained significant (*P* = 0.046). There was no difference in cold detection threshold (30.6°C [23.7—13.2°C] vs 30.6°C [26.8—31.4°C], *P* = 0.73, Mann–Whitney test).

### 3.3. Titration of thermal stimulation and task difficulty

The percept calibrated high (painful) thermal stimulus to be used during fMRI was set at a lower temperature for the patients with fibromyalgia, which was in keeping with thermal hyperalgesia identified by baseline QST. The temperature eliciting a pain intensity rating of 6 of 10 was 42 ± 2°C for patients with fibromyalgia and 43.1 ± 1.7°C for healthy controls (*P* = 0.047, Fig. 1E). The difficulty of the hard RSVP task to be used during the experiment was individually calibrated for each participant. Patients with fibromyalgia required a longer interstimulus interval in the RSVP task to perform at 70% of optimal (fibromyalgia: 96 ms [48—256 ms] vs control: 64 ms [32—96 ms], *P* = 0.008, Mann–Whitney test, Fig. 1F).

### 3.4. Pain ratings during the functional magnetic resonance imaging experiment

**Figure 2.**
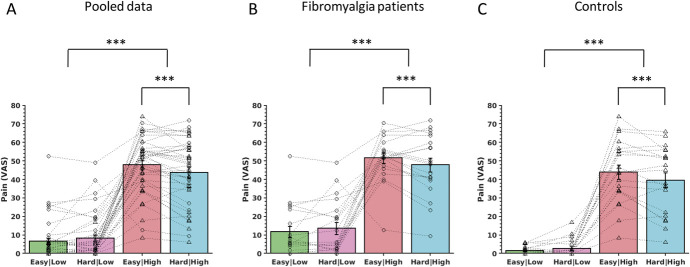
Pain ratings during the attentional analgesia experiment. (A) Pain ratings for each subject across experimental conditions (easy and hard task and low and high temperatures) pooled across groups (n = 40), and the same data are shown (B) split into patients with fibromyalgia (n = 20) and (C) healthy controls alone (n = 20). Mixed ANOVA showed a main effect of temperature and a task × temperature interaction mediated by a reduction in the pain scores in the hard|high condition (planned post-hoc paired *t* test). Mean ± SEM. (***P < 0.001). ANOVA, analysis of variance.

The objective of the experiment was to examine whether the pain evoked by the thermal stimuli (low or high temperature) was affected by the concurrent performance of the RSVP attention task (easy or hard task). The behavioural data (pain scores) were initially pooled for both groups (Fig. 2A). There was an expected main effect of temperature (F(1,38) = 174.8, *P* < 0.001, mixed ANOVA) and a temp × task interaction (F(1,38) = 13.1, *P* = 0.001, mixed ANOVA). There was no main effect of task (F(1,38) = 2.6, *P* = 0.12). A planned post-hoc analysis showed reduced pain in the hard|high (43.8 ± 2.8) vs the easy|high (47.9 ± 2.4) condition consistent with an attentional analgesic effect (*P* = 0.001, paired *t* test).

In the pooled analysis, there were no differences between the control and fibromyalgia groups (temp × group (F(1,38) = 0.2, *P* = 0.65), task × group (F(1,38) = 4.7, *P* = 0.66), or temp × task × group (F(1,38) = 0.01, *P* = 0.97)). To illustrate the behavioural similarity between the control group and the patients with fibromyalgia, the results are plotted separately (Figs. 2B and C). In healthy controls, a main effect of temperature and a task × temp interaction was evident (F(1,19) = 104.2, *P* < 0.0001 and F(1,19) = 11.9, *P* = 0.003, respectively). Similarly, in patients with fibromyalgia, there was a main effect of temperature and a task × temp interaction (F(1,19) = 73.9, *P* < 0.0001 and F(1,19) = 4.6, *P* = 0.046, respectively). For both groups, post-hoc paired t tests revealed that the interaction was due to an attentional analgesic effect with a decrease in pain scores in the hard|high vs the easy|high condition.

### 3.5. Task performance in the functional magnetic resonance imaging experiment

**Figure 3.**
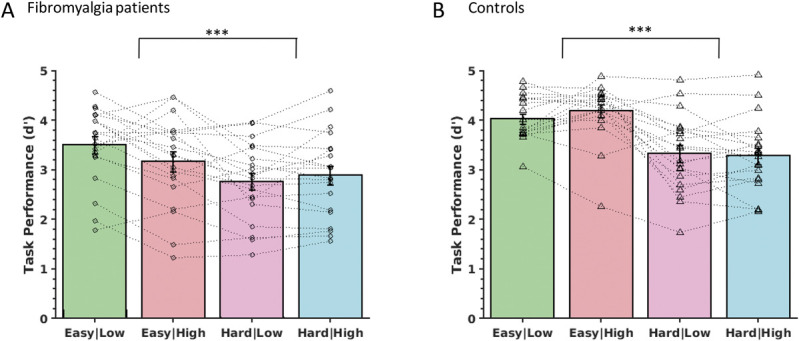
Task performance (d') in the scanner showing that the hard task was more challenging than the easy task for both (A) patients with fibromyalgia and (B) healthy controls. Mean ± SEM (mixed ANOVA, main effect of task ***P < 0.001). ANOVA, analysis of variance.

To assess performance on the RSVP task during the fMRI experiment, the subject's button responses were recorded and used to calculate d’. We noted that controls performed the task better overall in the scanner as reflected in the between-subject (ie, group) effect (F(1,38) = 10.2, *P* = 0.003), indicating that our initial calibration (outside the scanner) did not fully compensate for the differences in performance levels between the groups when they were challenged within the scanner (Fig. 3). Importantly, and as intended, the hard task was more challenging than the easy task with both groups showing a main effect of task (F(1,38) = 46.0, *P* < 0.0001, mixed ANOVA, Fig. 3). Patients with fibromyalgia and controls showed a similar drop in performance when comparing the easy with hard tasks because there was no interaction between task performance and group (F(1,38) = 2.7, *P* = 0.11). Further analysis indicated that stimulus temperature had no effect on task performance (main effect of temperature F(1,38) = 0.2, *P* = 0.63), and there was no interaction between task and temperature (F(1,38) = 0.9, *P* = 0.34) nor between temperature and group (F(1,38) = 2.6, *P* = 0.12).

### 3.6. Neuroimaging analysis

The behavioural results indicated that the patients with fibromyalgia had thermal hyperalgesia and overall a lower level of performance on the RSVP task, but when these factors were mitigated by adjusting stimulus temperature to percept and task speed to performance (in the prescanner session), they could still produce attentional analgesia. However, it was not clear if they recruited the same brain networks as healthy controls to produce this analgesic effect. Therefore, the same analysis strategy used for the behavioural pain ratings was also applied to the fMRI data. To determine main effects in the patterns of activation in the brain and the brainstem, data from both groups were pooled and subsequently differences between the subject groups were explored.

**Figure 4.**
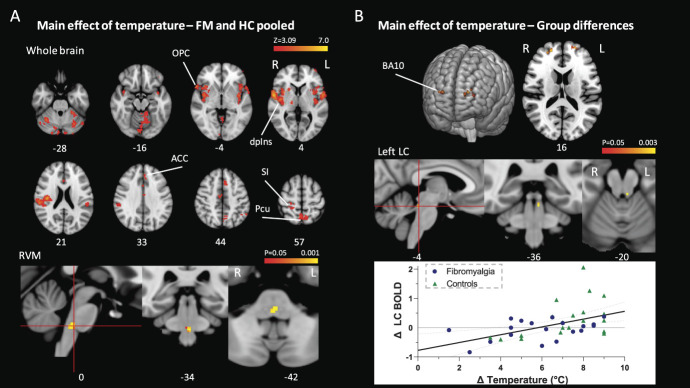
(A) Main effect of temperature in patients with fibromyalgia and healthy controls in the whole brain, showing activity in the dorsal posterior insula (dpIns), anterior cingulate cortex (ACC), and primary somatosensory cortex (S1) among others (Z > 3.1 cluster-corrected *P* < 0.05), and in the rostral ventromedial medulla (RVM, TFCE corrected *P* < 0.05). (B) Group difference in the main effect of temperature in the whole brain showing a stronger response in healthy controls in both Brodmann area 10 (BA10, Z > 3.1 cluster-corrected *P* < 0.05) and in the left locus coeruleus (LC, TFCE corrected *P* < 0.05). The correlation between main effect of temperature in LC and difference in temperatures between low and high (Pearsons *R* = 0.49, *P* = 0.002, dotted 95% confidence interval). OPC, operculum; Pcu, precuneus; TFCE, threshold free cluster enhancement.

Whole-brain analysis of the main effect of temperature in pooled group data revealed an expected pattern of activity in the forebrain regions commonly seen in pain imaging studies including prominent clusters in the contralateral (ie, right) dorsal posterior insula, primary somatosensory cortex, and anterior cingulate cortices (Fig. 4A and Table 2). Brainstem region–masked analyses showed a main effect of temperature in the RVM (Fig. 4A). Analysis of group-level differences in the whole-brain response to temperature showed no differences bar the singular exception of an enhanced response in healthy controls in the frontopolar cortex (Brodmann area 10, Fig. 4B and Table 2). Similar analyses in the brainstem showed a group-level difference in the left LC, again with an enhanced response in healthy controls (Fig. 4B). (Imaging data are available at: https://identifiers.org/neurovault.collection:9513.)

**Table 2:** Results from main effect analyses in the whole brain (cluster-forming threshold Z > 3.09 and cluster-corrected *P* < 0.05.)

Voxels	Z Max	X (mm)	Y (mm)	Z (mm)	Atlas labels
Main effect of temperature pooled groups					
2676	7	42	−12	8	83% central opercular cortex
1605	4.86	0	−74	−14	100% vermis VI
1292	6.09	−36	4	8	66% central opercular cortex
238	4.58	2	−62	54	69% precuneus cortex
166	4.87	24	−40	70	39% superior parietal lobule and 33% postcentral gyrus
156	4.19	−20	−84	−38	100% left crus II
121	4.48	0	30	28	70% cingulate gyrus, anterior division and 13% paracingulate gyrus
90	4.23	−54	−30	18	70% parietal operculum cortex; 6% central opercular cortex; 6% supramarginal gyrus, anterior division; and 5% planum temporale
85	4.81	−48	−66	−30	81% left crus I
84	4.53	−4	22	44	78% paracingulate gyrus and 7% superior frontal gyrus
79	4.73	2	−10	44	73% cingulate gyrus, anterior division and 17% cingulate gyrus, posterior division
77	4.16	−50	44	−10	83% frontal pole
73	3.85	−20	−88	−24	13% occipital fusiform gyrus and 66% left crus I
72	4.11	16	−14	6	97% right thalamus
65	4.34	30	−26	62	39% postcentral gyrus and 27% precentral gyrus
62	3.93	4	−6	12	34% left thalamus
62	4.71	−28	−50	−48	70% left VIIIa and 14% left VIIb
58	3.98	−38	62	8	54% frontal pole
56	3.77	−54	−52	48	46% angular gyrus; 33% supramarginal gyrus, posterior division; and 5% lateral occipital cortex
Group differences in main effect of temperature (controls > patients)					
124	3.98	−22	60	18	71% frontal pole
58	3.87	20	54	16	45% frontal pole
Main effect of task—pooled groups					
4234	6.22	−30	−94	8	5% lateral occipital cortex
3671	6.68	34	−86	4	21% lateral occipital cortex, inferior division
1147	6.27	8	28	30	48% paracingulate gyrus and 22% cingulate gyrus, anterior division
887	5.47	32	24	2	54% frontal operculum cortex, 11% inferior frontal gyrus and pars opercularis, and 5% inferior frontal gyrus and pars triangularis
382	5.53	−30	28	−2	54% insular cortex
273	5	−48	0	32	43% precentral gyrus, 12% middle frontal gyrus, and 11% inferior frontal gyrus and pars opercularis
182	4.03	−4	−42	−20	43% left I-IV
156	4.26	28	−52	54	43% superior parietal lobule and 12% angular gyrus
155	4.96	−8	−70	−16	98% left VI
140	5.27	4	−30	−4	70.9% brainstem
130	4.59	−54	−20	2	51% planum temporale and 10% Heschl gyrus (includes H1 and H2)
104	4.25	−8	−74	−38	64% left crus II and 31% left VIIb
54	3.75	−24	−68	−54	92% left VIIb
Negative main effect of task—pooled groups					
691	4.59	−6	−60	30	62% precuneus cortex
360	4.7	−38	−72	46	71% lateral occipital cortex, superior division
248	4.95	52	−62	42	66% lateral occipital cortex, superior division and 15% angular gyrus

The tables were created with Autoaq (part of FSL), with atlas labels based on the degree of overlap with probabilistic atlases (Harvard Oxford Cortical Structural Atlas, Harvard Oxford Subcortical Structural Atlas, and Cerebellar Atlas in MNI152 space after normalization with FNIRT). Only those structures to which the cluster had a >5% chance of belonging to are presented.

To explore the possible origins of these differences, we conducted an exploratory analysis based on the observed need to use a hotter high-temperature stimulus for the healthy controls than for patients with fibromyalgia (Fig. 1E). Therefore, the correlation of BOLD signal change for each area (BA10 and LC) and difference between the high-applied and low-applied temperatures was calculated. The left LC BOLD signal showed a positive correlation with the difference between high and low temperatures (Pearson *R* = 0.48, *P* = 0.02, Fig. 4B), suggesting that the difference in applied temperature might account for the group-level difference. A similar analysis did not reveal any correlation between temperature delta and activity in BA10 (*R* = 0.19, *P* = 0.47).

**Figure 5.**
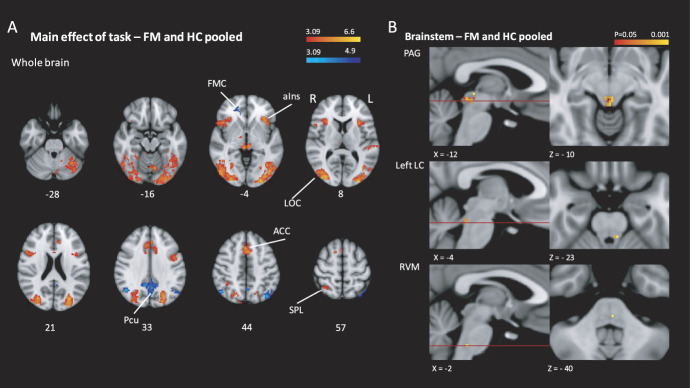
Main effect of task. Main effect of task in the pooled data from patients with fibromyalgia and healthy controls (A) in the whole brain, showing increased activity in the lateral occipital cortex (LOC), anterior insula (aIns), and anterior cingulate cortex (ACC) (red–yellow) and a decrease in activity in the precuneus (Pcu), lateral occipital cortex, and the frontomedial cortex (FMC, Z > 3.1 cluster-corrected *P* < 0.05). (B) Main effect of task in the brainstem: in the periaqueductal gray (PAG), RVM, and left LC (TFCE corrected *P* < 0.05). PAG, periaqueductal gray; RVM, rostral ventromedial medulla; SPL, superior parietal lobule. TFCE, threshold-free cluster enhancement.

Whole-brain analysis of the main effect of task in the pooled data showed a familiar pattern of increased activity in the visual attention network including the lateral occipital cortex, superior parietal lobule, anterior insula, and anterior cingulate cortex and a decrease in activity in the precuneus and fronto-medial cortex (Fig. 5A and Table 2). Brainstem region–masked analyses showed a main effect of task in the PAG, RVM, and left LC (Fig. 5B). No difference between the fibromyalgia and control groups was detected in the main effect of task at the whole-brain or brainstem level. (Imaging data are available at: https://identifiers.org/neurovault.collection:9513.)

No task × temperature or task × temperature × group interaction (that could be the neural substrate of the observed behavioural interaction between task and temperature, ie, attentional analgesia) was seen at the whole-brain or brainstem level.

**Figure 6.**
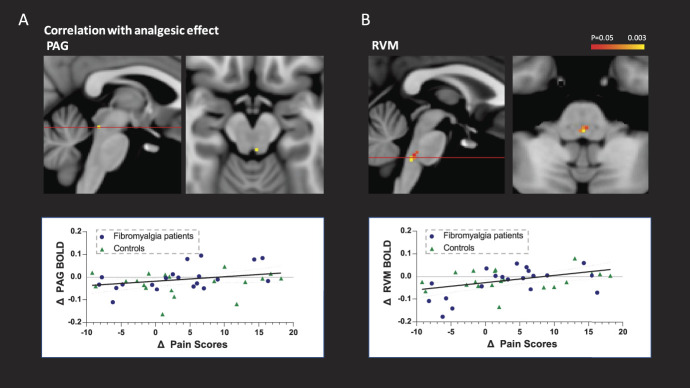
Direct relationship between BOLD and analgesia. Activity in the PAG and the RVM correlates with the attentional analgesic effect. Inter-subject parametric regression between BOLD in PAG and RVM with the analgesic effect (ie, delta pain ratings of easy|high—hard|high), (*P* < 0.05, TFCE corrected). PAG, periaqueductal gray; RVM, rostral ventromedial medulla; TFCE, threshold-free cluster enhancement.

A planned analysis sought correlations between the fMRI data (individual BOLD differences between hard|high and easy|high conditions) and the change in pain scores (ie, analgesic effect, easy|high minus hard|high) to improve the power to identify possible neurobiological substrates of the analgesic effect.^9,59^ The whole-brain regression analysis (ie, inter-subject) did not identify any significant regions showing correlation. However, masked brainstem analyses with the same model showed a positive correlation between analgesic effect and the change in activity in both the PAG and the RVM (Fig. 6).

## 4. Discussion

In this study, we demonstrate that, contrary to our expectation at the outset, patients with fibromyalgia can produce attentional analgesia with similar efficacy to healthy volunteers. Analysis of the pain ratings during the fMRI experiment revealed that diversion of attentional focus attenuated the pain reported in response to a hot thermal stimulus. This result is in contrast to previous evidence of malfunctioning endogenous pain modulation in patients with fibromyalgia.^42,44,48,74,80^ The specific exemplar of conditioned pain modulation has consistently been found to be impaired in patients with fibromyalgia,^8,32,62,65,71^ up to the point of becoming a test used for the evaluation of novel pharmaceutical therapies.^86^ It should be noted, however, that 2 previous reports have provided some evidence that attentional analgesia may be preserved in patients with fibromyalgia. Evoked pain was decreased while performing a Stroop task,^22,51^ although neither study was able to show significant difference in pain scores (ie, analgesia) between the easy (congruent) and hard (incongruent) version of the Stroop task. By controlling for task performance, we can identify that it is the cognitive task difficulty that is modulating pain percept and so demonstrates that this form of attentional pain modulation is intact in patients with fibromyalgia.

Other types of endogenous pain modulation such as placebo and music also produce some pain relief in patients with fibromyalgia,^23,27,34,61^ although with lower efficacy in patients with a longer disease duration.^45^ It therefore seems that cognitive modulation of pain more generically is functional in patients with fibromyalgia, and this may be a point of difference with conditioned pain modulation which is mediated by more of a hindbrain mechanism without a need for cortical drive. It has been proposed that the lack of analgesia induced by exercise or by a conditioned stimulus in patients with fibromyalgia is caused by the engagement of pain facilitatory networks.^32,41,48^ Another possibility is that the cortex–brainstem–spinal cord modulatory system is disrupted in patients with fibromyalgia and that they are only able to achieve analgesia by forebrain processes. The latter hypothesis was motivated by the finding of unchanged spinal withdrawal reflex during placebo analgesia, despite the reduction in pain scores, suggesting that the spinal cord activity was not modulated;^27^ however, this is at odds with other studies of placebo analgesia in healthy volunteers that have demonstrated a clear spinal modulation using fMRI.^18,19^ These contrasting findings with placebo analgesia raise the question of whether attentional analgesia in patients with fibromyalgia is mediated by engagement of descending control mechanisms as has been reported in healthy subjects.^9,59^

To resolve the brain regions involved in attentional analgesia, we used the same brainstem optimised imaging strategy as in our previous studies^9,59^: The analgesic effect in both groups correlated with the BOLD change PAG and RVM. This showed a positive linear relationship with the analgesic effect and suggests that these regions are mediating attentional analgesia. This is consistent with the proposition that patients with fibromyalgia can indeed recruit the descending pain modulatory system to generate attentional analgesia. Conclusive, direct evidence that PAG and RVM modulate the spinal cord during attentional analgesia is not yet present, but it has been repeatedly suggested.^9,59,73,76^ Functional imaging of the brainstem and spinal cord during an endogenous analgesia paradigm would help clarifying this issue by determining whether attentional analgesia is mediated by descending control from the brainstem to spinal cord to regulate nociception.

Quantitative sensory testing revealed thermal hyperalgesia in patients with fibromyalgia in response to both hot and cold stimuli, which is similar to that previously reported by other research groups.^5,8,36,65^ We also saw an apparently conflicting small increase in warm detection threshold without a change in cold detection threshold in patients with fibromyalgia. These slightly contradictory findings could fit with the proposition that this is due to altered functioning in the primary afferents because of a latent small fibre neuropathy^17,77^ and hyperexcitable C-nociceptors.^72^ On the other hand, recent evidence from a laser-evoked potential study failed to reveal the expected abnormal responses in patients with fibromyalgia.^2^ In our study, by carefully percept locking our thermal stimuli, we took account of the altered sensitivity between the groups, and the data from our imaging protocol do not shed any further light on this ongoing debate.

An alternative hypothesis regarding the aetiology of fibromyalgia is that the hyperalgesia is due to altered central processing.^12,15,30,66^ In support of this idea, it is worth noting that aberrant sensitivity is found in patients with fibromyalgia in many body locations and across sensory modalities (eg, thermal and mechanical pain^5^). Our results indicate that patients with fibromyalgia show a similar pattern of brain activation to the healthy controls in response to the percept-matched thermal stimulus (like Gracely et al.^30^), and there was no group difference in BOLD in response to task difficulty. However, we did find a difference between patients with fibromyalgia and controls in the anterior prefrontal cortex (BA10) and in the left LC in the main effect of temperature. Interestingly, the BOLD change in the LC correlated with the temperature used for the attentional analgesia experiment. Animal and human studies have shown that the LC is activated by noxious thermal stimuli.^35,59^ A similar relationship between human LC activity and thermal stimulus intensity has been made using pupillometry in healthy subjects.^20^ Therefore, it is possible that the difference in LC activity in this contrast is due to the patients receiving a significantly lower temperature compared with controls (to achieve the same pain score). On the other hand, the BOLD signal difference between the groups in BA10 does not correlate with applied temperature but is possibly related to cognitive aspects of pain perception.^63^ This region has been found to consistently respond to painful stimulus in healthy volunteers using a variety of imaging modalities (eg, fMRI, NIRS, and PET^63^), and it was reported that patients with fibromyalgia show reduced gray matter density in this area and in adjacent cortical regions.^47^ We also note a previous study comparing the response with pressure stimulation showed an area that was more active in control subjects than in patients with fibromyalgia that includes BA10.^30^ In addition, gray matter density in this area was reported to correlate negatively with the intensity of chronic pain.^25,46,54,57^ Thus, this region is hypothesized to be important in the chronification of pain, although its role in this context is yet to be fully elucidated.^63^ Therefore, the difference between the groups in BA10 activity in our study may well relate to the impact of an ongoing level of spontaneous pain (chronically present) seen in the patients with fibromyalgia that is not seen in the healthy controls. Our experimental design cannot demonstrate whether this is causally related. Overall, our findings do not provide evidence of substantial abnormalities in central pain processing in the fibromyalgia group and indeed show that the nociceptive processing as well as the engagement of descending control centres has many similarities.

We calibrated the hard version of the attentional task for each participant with the objective of achieving comparable cognitive load within and between groups (as per our previous studies^9,59^). We found that the intercharacter presentation interval was significantly longer in the fibromyalgia group compared with healthy controls. This is in line with previous findings reporting prolonged reaction times in the fibromyalgia group in, for example, a Stroop task^51,79^ and supports the evidence of impaired attentional/cognitive processes in patients with fibromyalgia. It has been proposed that such behavioural impairments are reflected by abnormal functioning of the caudate nucleus and hippocampus,^51^ a finding that is not reproduced in this study, which is to be expected because we adjusted task difficulty between the groups to produce equivalent performance which would mask any differences. Interestingly, however, during the experimental phase, patients with fibromyalgia performed worse than controls. This result may be consistent with the observation that painful stimulation has a disruptive impact on the cognitive ability of patients, possibly because of hypervigilance and catastrophizing.^2,14,22,28^ Nevertheless, it is important to note that even during the experiment, a contrast in performance between easy and hard task was present in patients with fibromyalgia. Indeed, the perceived difference in difficulty between the hard and easy task was homogeneous between groups, as evidenced by the absence of group difference in the main effect of task and both cohorts engaged the attentional network to a comparable degree.

A limitation of this study is that we were not able to precisely age match the control subjects with patients with fibromyalgia and by chance ended up with a significantly younger control group (by 8 years on average). Exploration of the influence of age, by inclusion as a covariate, in the analysis of the main effects of task and temperature and their interaction on pain scores, the heat and cold pain thresholds, and task performance in experiment did not substantially change the significance of any of our findings, so we do not believe that the difference in ages between the groups accounted for our findings.

In conclusion, this study demonstrates that patients with fibromyalgia are able to produce analgesia when engaged in a task that diverts their cognitive focus from a noxious stimulus. To this end, they engage brainstem nuclei in the same manner as healthy controls. This new evidence suggests that, contrary to what was believed, at least some of the elements of the descending pain modulatory system are functional in patients with fibromyalgia and are available to be recruited. This also lends weight to the idea that therapeutically encouraging patients with fibromyalgia to participate in cognitively engaging activities (as part of a multimodal rehabilitation package) may represent a useful therapeutic strategy because it may both aid their cognitive function and engage their descending pain control circuits to prioritise task performance.

## Conflict of interest statement

The authors have no conflicts of interest to declare.

## Supplemental video content

A video abstract associated with this article can be found at http://links.lww.com/PAIN/B376.

## Supplementary Material

SUPPLEMENTARY MATERIAL
